# SUMOylation inhibition enhances multiple myeloma sensitivity to lenalidomide

**DOI:** 10.1038/s41417-022-00450-9

**Published:** 2022-03-25

**Authors:** Li Du, Wei Liu, Flavia Pichiorri, Steven T. Rosen

**Affiliations:** 1 Toni Stephenson Lymphoma Center, Beckman Research Institute of City of Hope, Duarte, CA USA; 2 Judy and Bernard Briskin Center for Multiple Myeloma Research, Beckman Research Institute of City of Hope, Duarte, CA USA; 3 Department of Hematology and Stem Cell Transplant, Beckman Research Institute of City of Hope, Duarte, CA USA; 4 Department of Hematology, Xiangya Hospital, Central South University, Changsha, China; 5 City of Hope Comprehensive Cancer Center, City of Hope National Medical Center, Duarte, CA USA

**Keywords:** Cancer therapeutic resistance, Myeloma

## Abstract

Despite the potent effect of lenalidomide (Len) in multiple myeloma (MM) treatment, patients develop Len resistance leading to progressive disease, demanding an urgent need to investigate the mechanisms mediating Len resistance. Our study identified SUMOylation as a potential mechanism regulating Len resistance in MM. Len-resistant MM cell line MMR10R presented much higher SUMO E1 (SAE2) expression and more global SUMOylation than Len-sensitive MM1S cell line. SUMOylation inhibition by using TAK-981, a novel and specific SUMO E1 inhibitor, significantly enhances myeloma sensitivity to Len in MM cell lines. Moreover, the enhanced anti-MM activity by TAK-981 and Len combination has been validated using primary relapsing MM patient samples. Overexpression of IRF4 and c-Myc is a major mechanism of Len resistance. Len showed limited effect on IRF4 and c-Myc level in Len-resistance cell line, but TAK-981 treatment reduced IRF4 and c-Myc expression in Len-resistant line and caused further decrease when combined with Len. We found SUMOylation inhibition decreases IRF4 at transcriptional and post-translational level. SUMOylation inhibition reduced DOT1L with decreased methylation of histone H3 lysine 79, to suppress IRF4 gene transcription. SUMOylation inhibition also reduced IRF4 protein level by enhancing degradation. Overall, our data revealed SUMOylation inhibition enhances Len sensitivity through downregulating IRF4.

## Introduction

Multiple myeloma is an incurable hematological malignancy, emerging from plasma cells. As the second most common blood cancer, MM accounts for 1% of all cancers and 10% of hematologic malignancies in the United States. Worldwide there are ~100,000 deaths each year caused by MM [1]. Despite several new drugs that have improved the survival of myeloma patients in the past decade, patients typically develop relapsed and/or refractory MM and long-term disease-free survival remains low [2]. The immunomodulatory drugs IMiDs play a pivotal role in the treatment of MM. Lenalidomide (Len) is one of the most widely used IMiD drug in combination with Dexamethasone and antibody-based MM therapy. Interferon regulatory factor 4 (IRF4) and pro-survival myelocytomatosis viral oncogene (c-Myc) are a critical pathway for MM cell growth and survival [3–5]. IKZF1 (Ikaros) and IKZF3 (Aiolos), two zinic finger transcriptional factors, bind and activate the IRF4 promoter, which in turn enhance the transcription of c-Myc. Len directly binds to an E3 ubiquitin ligase Cereblon (CRBN), which rapidly triggers the ubiquitination of IKZF1/3, leading to degradation by proteasome [6–9]. Len mediated IKZF1/3 degradation leads to reduced IRF4 and MYC expression in MM cells and to loss of their viability.

Although majority newly diagnosed patients respond to Len therapy, most eventually develop resistance [10, 11]. Low CRBN expression was the first described mechanism associated with Len resistance in MM [12–14]. Other Len resistance mechanisms could bypass CRBN-IKZF1/3 axis to promote MM cell survival through upregulating pro-survival factors- IRF4 and c-Myc. The overexpression of IRF4 and c-Myc has been reported mediating Len resistance in MM [3–5]. Besides IKZF1/3, there are other transcriptional factors of IRF4 have been identified. DOT1 Like Histone Lysine Methyltransferase (DOT1L), which catalyzes methylation of histone H3 lysine 79, has been reported to be required for myeloma cell survival through enhancing IRF4-Myc signaling [15]. Transcription factor PU.1, encoded by gene SPI1, acts as tumor suppressor for myeloma cells through direct transcriptional repression of IRF4 [16, 17]. Despite all these findings, there is still urgent need to elucidate novel pathways involved Len resistance to develop new agents to enhance Len sensitivity [18–20].

One potential mechanism to address this need might be SUMOylation, a post-translational modification characterized by covalent attachment of small ubiquitin-like modifier (SUMO) proteins to a lysine (Lys) residue on target proteins [21–23]. It is carried out via an enzymatic cascade involving the sequential action of an activating enzyme E1 (a heterodimer of SAE1 and SAE2), a conjugating enzyme E2 (UBC9), and a ligating enzyme E3 (one of ~10). SUMOylation enzymes are expressed at higher levels in cancer cells than in normal cells; their elevated expression is required for tumor progression, cancer metastasis, and cancer stem cell maintenance and self-renewal, and is usually associated with poor survival in various human cancers, including MM, colorectal (CRC), and breast cancers [24–27].

In the present study, we found that the expression level of SUMO E1 (SAE2) and global SUMOylation were significantly higher in Len-resistant MM cell line compared to the parental Len-sensitive MM cell line MM1S. SUMOylation inhibition by using a novel selective SUMO E1 inhibitor, TAK-981, was effective against Len-sensitive and Len-resistant MM cell lines and primary relapsing MM samples. More importantly, the effect of TAK-981 was further enhanced in combination with Len. Further experiments indicated that TAK-981 treatment decreased key pro-survival factors IRF4 and c-Myc level. Therefore, we investigated how SUMOylation regulates IRF4-Myc pathway in MM.

## Materials and methods

### Reagents

TAK-981 was purchased from ChemieTek (IN, USA). Lenalidomide (SML2283) was purchased from Sigma (MO, USA).

### Multiple myeloma cell lines and primary samples

Primary MM cells were isolated from bone marrow aspirates of MM patients, using Ficoll-Hypaque density gradient sedimentation followed by CD138 microbeads separation (Miltenyi Biotec), with informed patient consent and the Research Ethics Board approval at City of Hope (IRB 15150). Normal B lymphocytes from healthy donor PBMCs were enriched by Mojosort human CD19+ cell selection kit (Biolegend) according to the manufacture manual. Purify was validated by flow cytometry of CD19 staining. Human myeloma cell lines MM1S, H929, KMS11, and RPMI8226 were obtained from ATCC. Lenalidomide resistant cell line MMR10R was a kind gift from Dr. R Z Orlowski (M.D. Anderson cancer cell, TX, USA) [28]. All myeloma cell lines and primary CD138+ MM cells were cultured in RPMI1640 medium (Corning) with 10% heat-inactivated FCS (Omega Scientific, Inc.), 2 mmol/L l-glutamine, and 1% antibiotic-antimycotic (Life Technologies). Mycoplasma was routinely tested using Mycoplasma PCR detection kit (G238, Abcam).

### MM cell line transfection

Plasmid MSCB-hDot1Lwt was a gift from Dr. Yi Zhang (Addgene plasmid # 74173; http://n2t.net/addgene:74173; RRID: Addgene_74173) [29]. MM1S cells were transfected by electroporation using Nucleofector 4D system and SE Cell Line 4D-NucleofectorTM X Kit L(Lonza). Briefly, 1×10^6^ cells were resuspended in 100 μl of the nucleofector solution SF, 3 μg of plasmid MSCB-hDot1Lwt, or empty control vector were added and transferred to a cuvette. Program CA-137 was used for MM1S cells. After electroporation, cells were immediately plated out in pre-warmed medium onto 12-well plate. Compound treatments were performed after 24 h.

### Cell viability assay and drug-synergy calculations

Cells (0.5–2 × 10^4^/200 μl/well) were cultured in 96-well plates and treated with the indicated reagents for 48 h or 72 h. Cell viability assays were performed using Cell Titer-Glo Luminescent Cell Viability (G7572, Promega) according to the manufacturer’s instruction. Median inhibitor concentration (IC_50_) was determined using GraphpadPrism 8.0. Combination indices (CIs) were calculated using CompuSyn software (Biosoft, Cambridge, UK). Simulating calculated CI values and experimental CI values based on combination data points are plotted as a function of the fraction affected (Fa). Fraction affected indicates percentage inhibition of cell, growth/100. Synergism, additive effect and antagonism of combine treatment assays are defined as CI < 1, CI = 1 and CI > 1 respectively, utilizing the Chou-Talalay Method [30, 31]. For MM1S and MMR10R cell line drug-synergy analysis, SynergyFinder software was used to calculate synergy scores using effect-based strategy, Highest Single Agent (HSA) model or dose-effect-based strategies, Loewe additivity model. Synergy scores > 0 indicate synergism (red regions) and scores < 0 indicate antagonism (green regions) [32].

### Flow cytometry-based apoptosis assay

Cell apoptosis was measured after Annexin V FITC and PI staining (# 556419, BD Bioscience) according to the manufacturer’s instructions using a BD Fortessa LSR II and FlowJo Version V10.6.2.

### Western blot

Cells were harvested and lysed in Laemmli sample buffer (5% SDS, 25% glycerol, 150 mmol/L Tris-HCl pH 6.8, 0.01% bromophenol blue). After protein concentration was measured using BCA protein assay, 0.7 mol/L β-mercaptoethanol was added and protein samples were boiled for 10 minutes. Samples were separated by SDS-PAGE, and protein was transferred onto a polyvinylidene fluoride membrane (Immobilon-P membrane, Millipore). Following antibodies were used: SAE2 (ab58451, abcam), SUMO-2,3 (M114-3, MBL), c-Myc (ab32072, Abcam), GAPDH (sc-20357, Santa Cruz Biotechnology), SUMO-1 (#4930), IRF4 (#4964), CRBN (#71810), cleaved PARP (#5625), Aiolos (#15103), Ikalos (#14859), DOT1L (#77087), H3K79me2 (#5427), PU.1 (#2266), UBC9(#4918) and SAE1(#13585) were from Cell Signaling Technology. Western blot results were visualized using an Odyssey detection system (Licor) or Pierce ECL Western Blotting Substrate (Thermo Fisher Scientific).

### Reverse Transcription and qPCR

Total cellular RNA was extracted using miRNeasy Mini Kit (Qiagen). Total RNA (2 μg) was reverse-transcribed using an Omniscript RT Kit (Qiagen) and oligo dT primer. Real-time qPCR of gene expression was performed using the SYBR-Green Master Mix (Applied Biosystems). All quantitative PCR reactions were performed using ViiA 7 real-time PCR system (Applied Biosystem). Relative expression was calculated using the comparative Ct method normalized to GAPDH. The following primers were used for PCR: IRF4 sense, 5′-GCTGATCGACCAGATCGACAG-3′; IRF4 antisense, 5′-CGGTTGTAGTCCTGCTTGC-3′; DOT1L sense, 5′-GAGACCTCCTTCGACCTGGT-3′; DOT1L antisense, 5′-CGACGCCATAGTGATGTTTGC-3′; GAPDH sense, 5′-AGGTCGGAGTCAACGGATTTG-3′; and GAPDH antisense, 5′-GTGATGGCATGGACTGTGGT-3′.

### Chromatin immunoprecipitation assay

To detect the binding occupancy of DOT1L or H3K79me2 on the human IRF4 promoter, chromatin immunoprecipitation (ChIP) analysis was conducted using SimpleChIP Enzymatic Chromatin IP kit (Magnetic Beads) (#9003, Cell Signaling Technology). A total of 2 × 10^7^ MM1S or MMR10R cells were incubated in culture medium containing 1% formaldehyde for 10 min at room temperature, after which, the cross-linking reaction was quenched with addition of glycine to a final concentration of 0.125 mol/L. Cells were washed with cold PBS and harvested, followed by sonication to produce chromatin of primarily mononucleosome size. Fragmented chromatin was then incubated with DOT1L or H3K79me2 antibody (#5427, Cell Signaling Tech) at 4 °C overnight. Protein–DNA complexes were recovered using protein G dynabeads, washed, and eluted with elution buffer. Crosslinks were reversed at 65 °C in 0.25 mol/L NaCl overnight; then, the DNA was digested with proteinase K for 2 h at 50 °C. The immunoprecipitated DNAs were subsequently isolated and used for qPCR. Primer for ChIP: forward, 5’-TTCGCATGCCATCTGTCATG-3’, reverse, 5’-TTTTCAGCAACTCCCTTGGG-3’

### Protein degradation assay

IRF4 protein stability was measured on treatment with protein synthesis inhibitor cycloheximide (CHX). Cells treated with 100 μg ml^−1^ CHX (#2112, Cell Signaling Technology, Inc.) were collected at different time points and cell lysate was used for western blot to determine the protein level at different CHX treatment time. Western blot results were quantified by the ImageJ Software (NIH).

### Gene expression analysis from public datasets

UBA2 and DOT1L expression extracted from GEO databases was plotted and analyzed.

### Statistical analyses

No samples were excluded from analysis. For all experiments, *P* values were derived using a two-tailed Student’s *t*-test or ANOVA. Data presented as mean ± SD. Estimated variation is indicated as SD in each figure. For all graphs, *p < 0.05, **p < 0.01 and ***p < 0.001.

## Results

### Expression of SUMO E1 is upregulated in Lenalidomide resistant MM cells

**Fig. 1:**
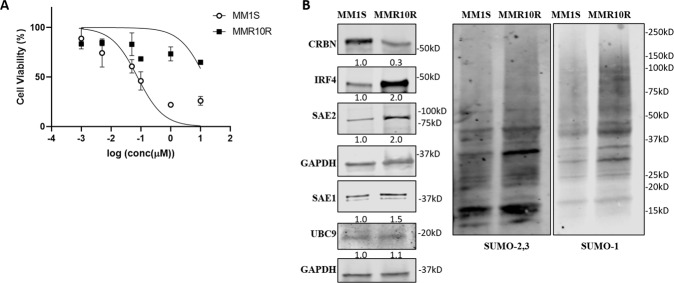
Lenalidomide resistant MM cells have higher SAE2 and global SUMOylation than Lenalidomide sensitive MM cells. **A** Cell viability assay showing Len-sensitive MM1S and Len-resistant MMR10R cell line treated with Lenalidomide at indicated concentrations. IC_50_ values were calculated by GraphPad Prism 8. **B** Western blot showing CRBN, IRF4, SAE2, SAE1, UBC9, and global SUMOylation (SUMO-2,3 and SUMO-1) level of MM1S and MMR10R cell lines; GAPDH, loading control. Relative protein level was quantified using Image J, normalized to GAPDH, and labeled below each blotting band.

MM cell line with acquired resistance to Len (MMR10R) was established by culturing MM1S with addition of Len to the medium for an extended period of time as previously described [28]. IC_50_ values of Len in MM1S and MMR10R were determined by cell viability assay. MMR10R cell line presented resistance to Len compared to MM1S, with IC_50_ at 15 vs 0.1 µM (Fig. 1A). Western blot indicated MMR10R cells showed a significant decrease of CRBN and a huge induction of IRF4 protein, which is consistent with previous reports that loss of CRBN and overexpression of IRF4 contribute to Len-resistance in MM. More importantly, MMR10R cells expressed greater levels of SUMO E1 SAE2, SAE1, and global SUMOylation (SUMO-1 and SUMO-2,3) than MM1S cells. SUMO E2 enzyme, UBC9 level showed no difference between these two cell lines (Fig. 1B). These results suggest SUMOylation, especially SUMO E1, might be involved in Len resistance mechanism.

### SUMOylation inhibition enhances Len anti-MM activity in cell lines and primary patient samples

**Fig. 2:**
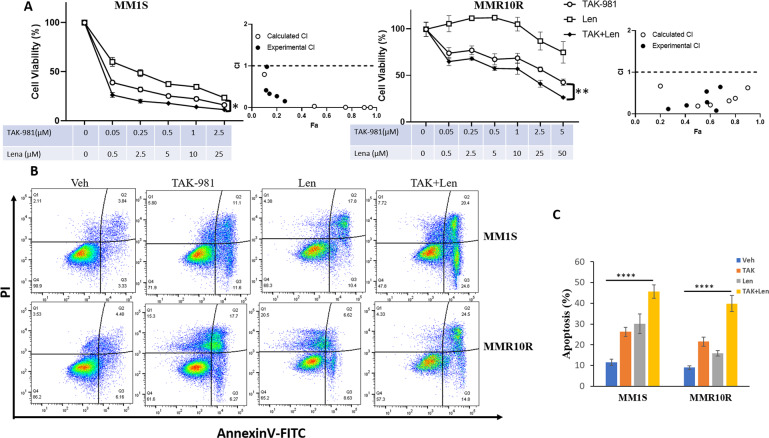
SUMOylation inhibition synergizes with Len in decreasing cell viability in both Len-sensitive and Len-resistant MM cell lines. **A** TAK-981 synergizes with Len cytotoxicity in sensitive MM line (MM1S) and resistant MM line (MMR10R). MM1S and MMR10R cells were treated with indicated concentration of TAK-981 or Len or both (TAK + Len) with indicated concentration for 48 h and cell viability was determined by Cell-Titer-Glo. Drug synergy was analyzed using CompuSym program. Simulating calculated CI values (open circle) and experimental combination indice (CI) values (solid circle) based on combination data points are plotted as a function of the fractional affected (Fa) derived from analysis report. Fraction affected indicates percentage inhibition of cell growth/100. Drug synergism is defined as CI < 1. **B** TAK-981 enhances cytotoxicity of Len in sensitive and resistant MM. MM1S and MMR10R cells were treated with Vehicle (Veh), 0.1 µM TAK-981 (TAK), 2.5 µM Len (Len), or 0.1 µM TAK-981 with 2.5 µM Len (TAK + Len). Apoptosis was measured by flow cytometry using Annexin V/PI staining. **C** Quantified apoptosis from three experimental repeats. Data were analyzed using one-way ANOWA test: Data presented as mean ± SD. *p < 0.05; **p < 0.01; ****p < 0.0001.

To test if inhibition of SUMOylation can sensitize the effects of Len in MM, we utilize TAK-981, a novel, selective small molecule inhibitor of SUMO E1 enzyme, which is currently in Phase 1 trials in adult patients with metastatic solid tumors and lymphomas [33]. We conducted cytotoxicity assay in MM1S and MMR10R cell lines, TAK-981 synergized with Len at decreasing cell viability in both Len-sensitive and Len-resistant cell lines with CI values calculated by CompuSyn (Fig. 2A). We also performed synergy matrix with TAK-981 and Len in MM1S and MMR10R cells. SynergyFinder software was used to calculate synergy scores using effect-based strategy, Highest Single Agent (HSA) model or dose-effect-based strategies, Loewe additivity model. Both models confirmed the synergistic effects of TAK-981 and Len in both cell lines MM1S and MMR10R (Supplementary Fig. S1). Len showed limited effects on inducing apoptosis or inhibiting cell growth in MMR10R. Annexin-V staining indicated TAK-981 treatment led to apoptosis in both cell line, and the effects were further enhanced in combination with Len (Fig. 2B, C). The synergistic effects of combination TAK-981 and Len were observed in other MM cell lines RPMI8226 and H929 (Supplemental Fig. S2).

**Fig. 3:**
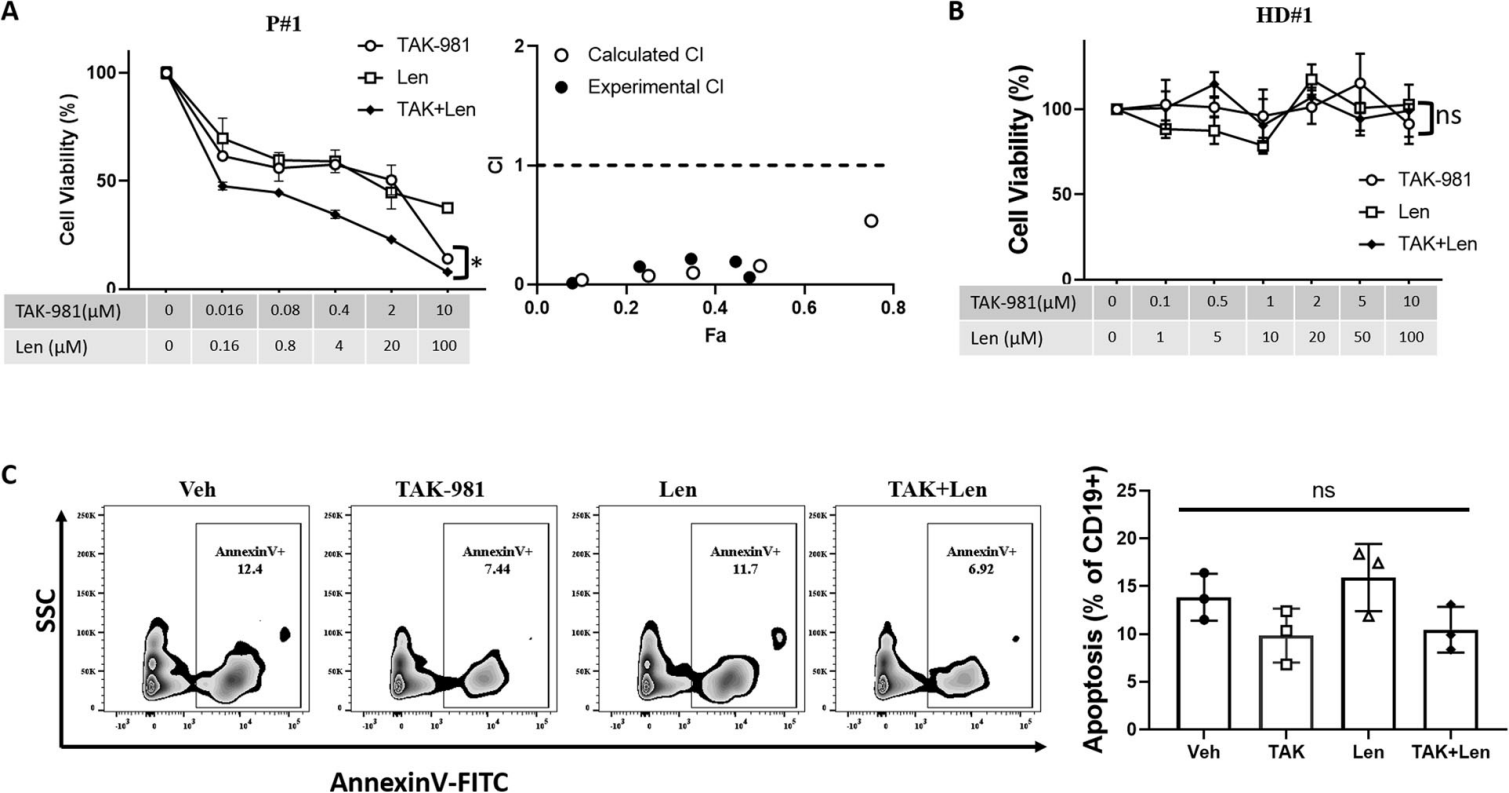
SUMOylation inhibition synergizes with Len in decreasing cell viability in primary multiple myeloma cells but doesn’t affect normal primary B lymphocytes viability. **A** Cell viability assay showing 1 out of 6 primary CD138+ cells from bone marrow aspirates of relapsing MM patients treated with TAK-981 or Len or both (TAK + Len) with indicated concentration. Cell viability was assessed by Cell-Titer-Glo after 48 h of treatment. Drug synergy was analyzed using CompuSym program. Combination Indice (CI) values are plotted. Drug synergism is defined as CI < 1. **B** Cell viability assay showing 1 out of 3 primary CD19+ cells from PBMCs of healthy donors. Normal B lymphocytes were purified by Mojosort human CD19+ cell selection kit. CD19+ cells were treated with TAK-981 or Len or both (TAK + Len) with indicated concentration. Cell viability was assessed by Cell-Titer-Glo after 48 hours of treatment. **C** TAK-981 or Len or both showed no effect on normal CD19+ cell viability by apoptosis assay. Healthy donor PBMC cells were treated with Vehicle (Veh), 0.1 µM TAK-981 (TAK), 2.5 µM Len (Len), or 0.1 µM TAK-981 with 2.5 µM Len (TAK + Len). Apoptosis was measured by flow cytometry using Annexin V staining gated on CD19+ population. Quantified apoptosis from three individual healthy donors was plotted. Data presented as mean ± SD. Data were analyzed using ANOVA test. Ns, not significant, *p < 0.05.

We then evaluated the effect of TAK-981 and Len combination using primary relapsing MM patient samples. CD138+ primary MM cells were isolated from relapsing myeloma patients and treated with TAK-981 alone, Len alone or both for 72 h, then cell viability was measured. Among 5 out of 6 primary MM samples, combination of TAK-981 with Len showed significantly enhanced cytotoxicity compared to the single agents alone in MM patient samples (Fig. 3A and Supplementary Fig. S3). The effects of TAK-981 on enhancing Len effects in MM cell lines and primary samples suggests SUMOylation inhibition might enhance MM sensitivity to Len.

To evaluate the effect of SUMOylation inhibition on non-transformed primary B lymphocytes, CD19+ cells were isolated from healthy donor PBMCs then treated with TAK-981 or Len for cell viability assay. There is no obvious cytotoxicity observed upon either TAK-981, Len or combination treatment in all three tested healthy donor PBMCs (Fig. 3B and Supplementary Figure S4AB). In PBMCs treated with TAK-981, Len or both, Annexin V staining in CD19+ gated cells were used to measure the apoptotic cell percentage. Consistent with Fig. 3B, no difference of apoptosis of CD19+ cells was observed in TAK-981, Len or combination treatment compared to vehicle control (Fig. 3C and Supplementary Fig. S4C). Cell viability assay of PBMCs indicated that TAK-981 or Len or both showed no effect on cell viability of health donor PBMCs (Supplementary Fig. S4D). Taken together, TAK-981 showed cytotoxicity on MM cells without affecting normal primary B lymphocytes cell viability.

### SUMOylation inhibition decreased IRF4 level independent of CRBN-IKF1/3 regulation

**Fig. 4:**
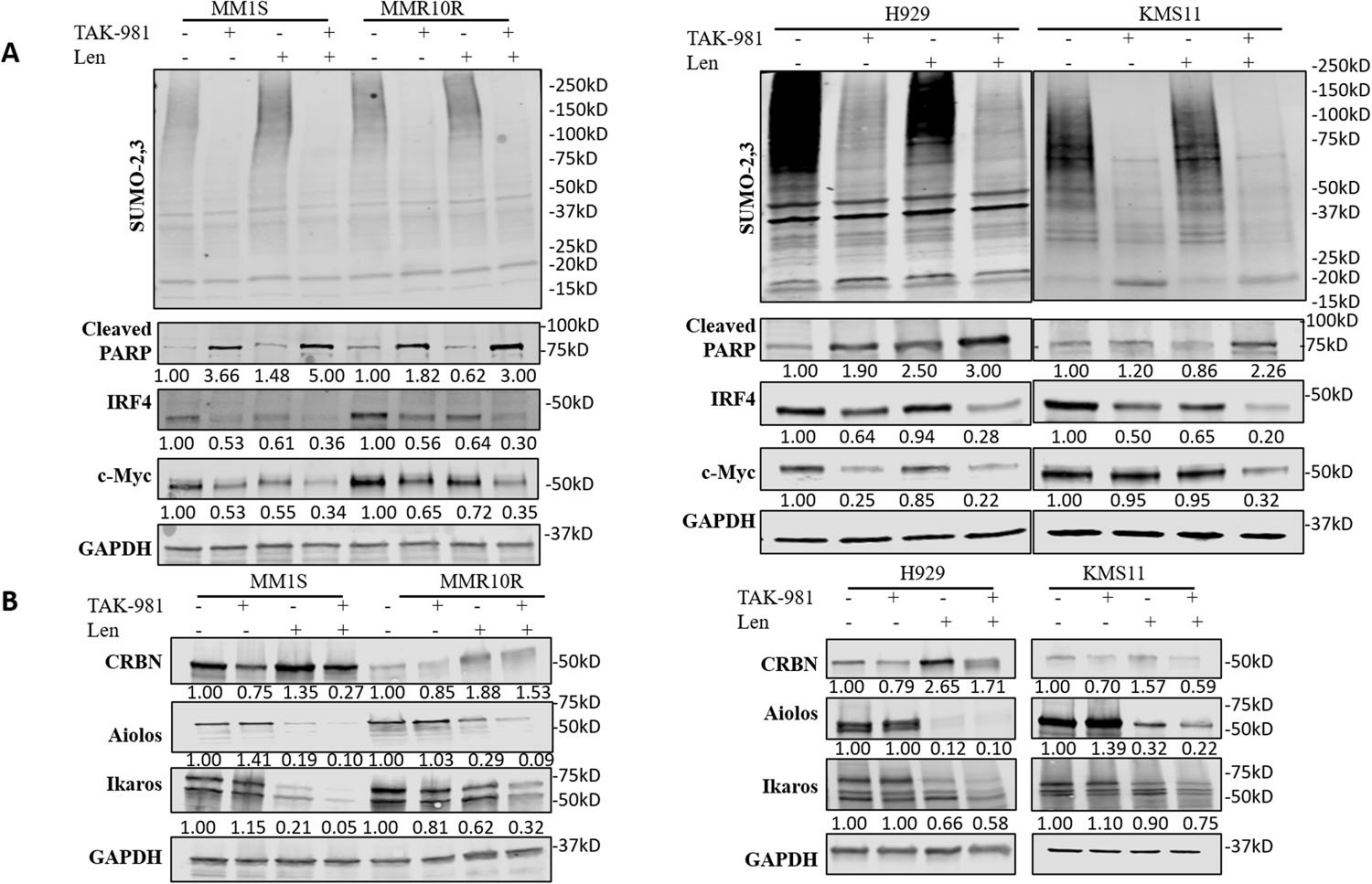
SUMOylation inhibition decreased IRF4 level independent of CRBN-IKF1/3 regulation. **A** TAK-981 synergizes with Len at increasing apoptosis marker cleaved PARP and decreasing IRF4 and c-Myc levels in MM. Western blot showing global SUMOylation (SUMO-2,3), cleaved PAPR, IRF4, and c-Myc level of MM1S and MMR10R (left) and H929 and KMS11 (right) cell lines. **B** Western blot showing CRBN, Aiolos (IKZF3) and Ikaros (IKZF1) level of MM1S and MMR10R (left) and H929 and KMS11 (right) cell lines. MM1S, MMR10R and H929 cells were treated with TAK-981 (0.1 µM) or Len 2.5 µM or both for 48 h; KMS11 cells were treated with TAK-981 (1 µM) or Len (25 µM) or both for 48 h. GAPDH was used as loading control. Relative protein level was quantified using Image J, normalized to GAPDH, and labeled below each blotting band.

To explore how SUMOylation inhibition mediates Len resistance, we evaluated the impact of TAK-981 on IRF4 and c-Myc, two key regulators mediating MM cell growth and Len resistance. 4 MM cell lines were treated with TAK-981 alone, Len alone or both for 48 h, cell lysates were collected for western blot. Len treatment caused downregulation of IRF4 and MYC with increased apoptosis marker cleaved PARP level, but the effects were limited in MMR10R cells (Fig. 4A). However, TAK-981 treatment significantly decreased IRF4 and c-Myc levels along with increased cleaved PARP expression in both MM1S and MMR10R cell lines. The decrease of TAK-981 on IRF4 and c-Myc level was further enhanced when combined with Len. Consistent effects were observed in other two MM cell lines H929 and KMS11(Fig. 4A). The data indicated SUMOylation inhibition enhanced Len effect at suppressing MM cell growth by downregulation of IRF4 and c-Myc expression. Then, CRBN-IKZF1/3 pathway was investigated. Len treatment significantly induced CRBN level followed by dimished expression of Aiolos and Ikaros in MM1S cells. But CRBN-mediated degradation of Aiolos and Ikaros upon Len treatment was substantially reduced due to loss of CRBN in MMR10R cells. TAK-981 treatment, although slightly decreased CRBN level, showed little effect on downstream protein levels of Aiolos and Ikaros. Consistent results were observed in H929 and KMS11 as well (Fig. 4B). These findings indicated SUMOylation inhibition decreased IRF4 level through a different regulation mechanism.

### SUMOylation inhibition decreased IRF4 level through downregulating DOT1L and H3K79me2 at IRF4 promoter region

**Fig. 5:**
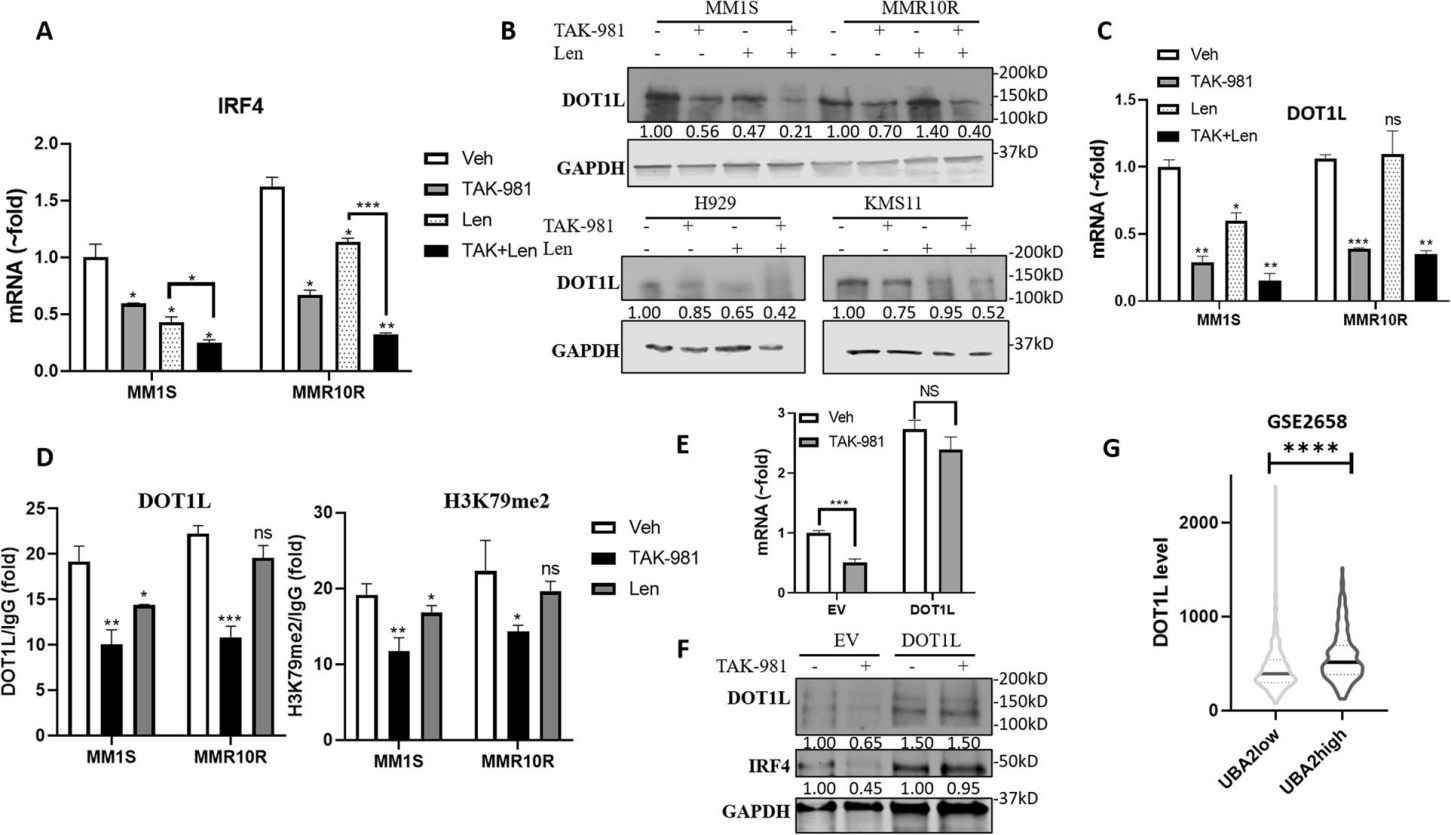
SUMOylation inhibition decreased IRF4 level through downregulating DOT1L and H3K79me2 at IRF4 promoter region. **A** TAK-981 synergizes with Len at decreasing IRF4 mRNA level in MM1S and MMR10R cell lines determined by quantitative PCR (qPCR). **B** Western blot showing decreased DOT1L level upon TAK-981 treatment in MM1S and MMR10R (Top) and H929 and KMS11 (Bottom) cell lines. Cells were treated as described in (Fig. 4). **C** TAK-981 treatment decreases DOT1L mRNA levels in both MM1S and MMR10R cell lines and Len has no effect on DOT1L level in MMR10R cell line measured by qPCR. **D** TAK-981 treatment decreased the occupancy of DOT1L and H3K79me2 on the IRF4 promoter region as measured by ChIP assay. ChIP was performed using an anti-DOT1L and H3K79me2 antibody in MM1S and MMR10R treated with TAK-981 (0.1 µM) or Len 2.5 µM for 48 h. The occupancy was normalized to DNA input and calculated relative to IgG control. **E** Overexpression of DOT1L compensated the decrease of IRF4 mRNA level caused by TAK-981 treatment. MM1S cells were transduced with plasmid MSCB-hDot1Lwt expressing DOT1L(DOT1L) or empty vector (EV) by electroporation then treated with TAK-981(0.1 µM) for 48 h. IRF4 mRNA level was determined by qPCR. Data presented as mean ± SD. ns, not significant; ***p < 0.001. **F** Overexpression of DOT1L compensated the decrease of IRF4 protein level caused by TAK-981 treatment. Western blot presenting IRF4 and DOT1L protein level in same treatment of (**E**). GAPDH, loading control. Quantified protein level was labeled below each blot. **G** UBA2 level correlates with DOT1L expression in patient specimens. Analysis of cohort (GSE2658) of 559 MM patients. Patients with high SAE2 (UBA2; UBA2high group) showed higher DOT1L level than patients with low SAE2 (UBA2; UBA2low group). Data were analyzed using unpaired Student *t* tests: Data presented as mean ± SD. ****p < 0.0001.

In order to evaluate the regulation of IRF4 at transcriptional level, IRF4 mRNA level was determined in MM1S and MMR10R cells by real-time qPCR. MMR10R cells exhibit higher IRF4 mRNA level than MM1S. Len treatment greatly decreased IRF4 mRNA in MM1S but showed less effect in MMR10R, but TAK-981 treatment decreased IRF4 mRNA level in both MM1S and MMR10R cell lines and showed further reduction in combination with Len (Fig. 5A). DOT1L has been reported to promote MM cell proliferation through activating IRF4 transcription via methylation of histone H3 lysine 79 at the promoter region. We found Len treatment reduced DOT1L level in MM1S, H929, and KMS11 cell lines but showed no effect in MMR10R cell line. TAK-981 treatment significantly reduced DOT1L level in all 4 MM cell lines and the reduction was further enhanced in combination with Len, even in Len-resistant MMR10R cell line (Fig. 5B). DOT1L mRNA level showed consistent change as protein level in MM1S and MMR10R cells upon TAK-981 and Len treatment (Fig. 5C).

We performed ChIP assay to determine the binding occupancy of DOT1L and H3K79me2 level at the IRF4 promoter region in MM1S and MMR10R cells treated with TAK-981 or Len. TAK-981 significantly decreased DOT1L occupancy and H3K79me2 level at IRF4 promoter at similar extent levels in both MM1S and MMR10R cell lines. Len showed less effect than TAK-981 in MM1S cells and barely no effect in MMR10R (Fig. 5D). We then investigated if the decrease of IRF4 level caused by TAK-981 treatment could be compensated by overexpression of DOT1L. We transfected plasmids expressing DOT1L or empty vector in MM1S cells by electroporation then treated MM1S cells with or without TAK-981 for 48 h, then measured IRF4 mRNA level by qPCR and protein level by western blot. TAK-981 treatment significantly decreased IRF4 mRNA level (1:0.5 fold) in empty vector transfected cells, but only slightly decreased IRF4 level (1:0.9fold) in cells with overexpression of DOT1L, indicating overexpression of DOT1L compensated the IRF4 level (Fig. 5E). Western bolt showed consistent results (Fig. 5F). The results suggest SUMOylation inhibition caused decrease of IRF4 is through mediating DOT1L.

We analyzed a MM patients data set GSE2658 (*n* = 559), among which SAE2(UBA2) level is associated with poor outcome [34]. Patients with high SAE2 (UBA2; UBA2high group) showed higher DOT1L level than patients with low SAE2 (UBA2; UBA2low group) (Fig. 5G), indicating the regulation of DOT1L expression by SAE2 is not restricted to cell lines. These results indicated SUMOylation inhibition decreases IRF4 level via downregulating the transcription activator DOT1L.

PU.1, an E-twenty-six family transcription factor, has been reported as a transcriptional factor suppressing IRF4 expression, acting as a tumor suppressor in MM [16]. We observed TAK-981 treatment increased PU.1 protein and mRNA levels in MM1S and MMR10R cell lines (Supplementary Fig. S5A, B). Analyzing MM patient cohort GSE2658 indicated SAE2 level was negatively associated with PU.1 (gene name SPI1) expression (Supplementary Fig. S5C), suggesting SUMOylation inhibition might increase PU.1 to decrease IRF4 expression. However, TAK-981 treatment didn’t show any change on PU.1 level in H929 and KMS11 cell lines (Supplementary Fig. S5D). Although TAK-981 and Len both increased PU.1 level in MM1S and MMR10R, the combination treatment led to no change or even less PU.1 level compared to vehicle treatment. The results are not consistent with our observation that combining TAK-981 and Len showed lower IRF4 level than single agent treatment, suggestion the regulation of PU.1 by SUMOylation may not contribute to the synergistic effect in TAK-981 and Len combination.

### SUMOylation inhibition affects IRF4 protein stability

**Fig. 6:**
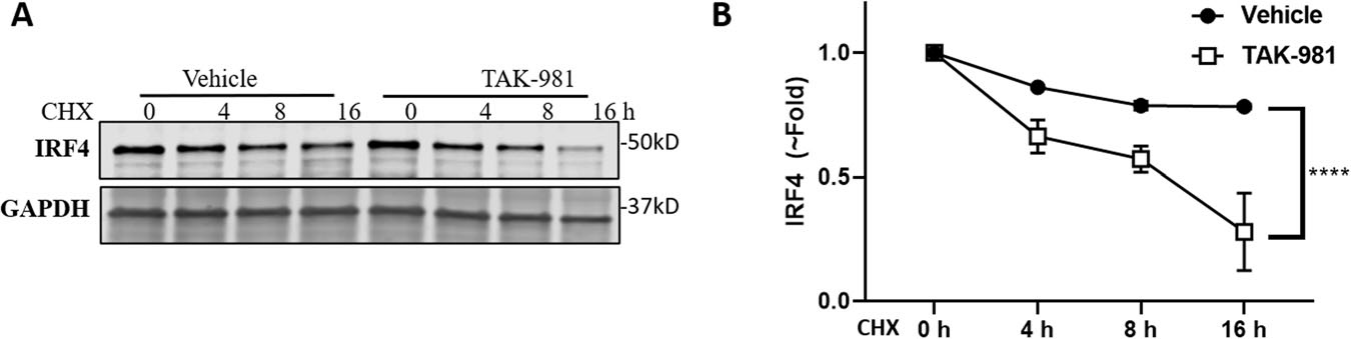
SUMOylation inhibition accelerates IRF4 protein degradation. **A** Representative western blot of IRF4 level over time in MMR10R cells treated with or without TAK-981(0.1 µM) for 4 h, followed by 100 μg ml^−1^ CHX treatment for indicated time; GAPDH, loading control. **B** IRF4 decay curve was determined by quantifying protein level normalized to GAPDH from three independent experiments.

IRF4 has been identified as SUMO target protein, which can be SUMO-modified at Lysine 349 (K349) by SUMO-2 [35]. SUMOylation can promotes IRF4 protein stability. We then evaluated the impact of TAK-981 on the protein stability using a cycloheximide (CHX) chase assay. MMR10R cells were treated with or without TAK-981 followed by addition of CHX to block protein synthesis, which allows monitoring protein degradation. Western blot showed IRF4 degraded faster in myeloma cells treated with TAK-981 compared to vehicle (Fig. 6 and Supplementary Fig. S6), indicating SUMOylation inhibition decreased IRF4 protein level through enhancing degradation.

## Discussion

Overcoming Lenalidomide resistance is a critical medical need in MM therapy. Identification of novel mechanism of Lenalidomide resistance and developing new agents to sensitize the effect of Lenalidomide is of the utmost importance. Our study uncovered a mechanism in which SUMOylation inhibition reduces IRF4 and c-Myc level, leading to enhancement of Len sensitivity in MM. SUMO E1 inhibitor TAK-981 was effective against Len-resistant cell line and primary relapsing MM samples. Moreover, combination TAK-981 with Len showed potent synergistic anti-MM effects, supporting translation of this SUMO E1 inhibitor into myeloma trials.

**Fig. 7:**
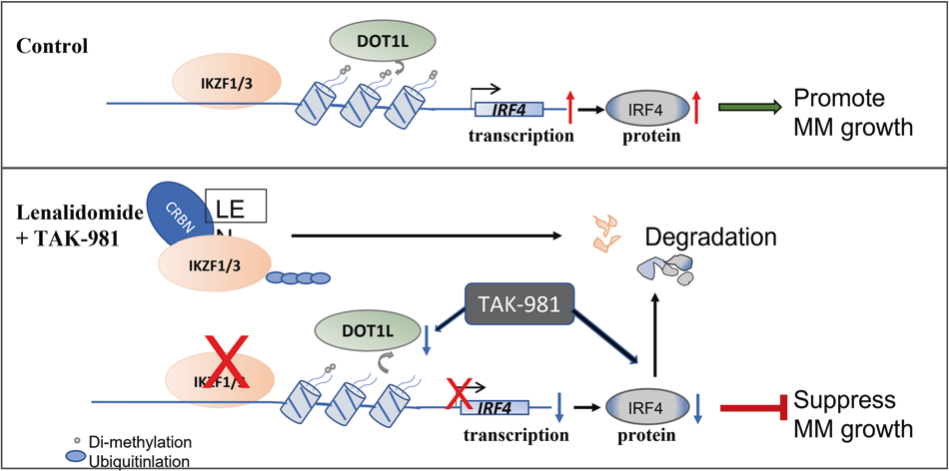
Schematic diagram showing the mechanism of how SUMOylation inhibition sensitizes Lenalidomide effects in MM cells. Upper panel (Control): Two transcriptional factors, IKZF1 (Ikaros) and IKZF3 (Aiolos), bind and activate the IRF4 promoter. Methyltransferase DOT1L can activate IRF4 transcription via methylation of histone H3 lysine 79 at the promoter region. Both IKZF1/3 and DOT1L enhance IRF4 expression, thus promote MM cell growth. Lower panel (Lenalidomide+TAK-981): Len directly binds to an E3 ubiquitin ligase Cereblon (CRBN), which rapidly triggers the ubiquitination of IKZF1/3, leading to degradation by proteasome. TAK-981 decreases DOT1L level and causes less DOT1L and H3K79me2 binding at IRF4 promoter region. Both drugs treatment leads to decreased transcription of IRF4 mRNA level. Further, TAK-981 treatment inhibits the SUMOylation of IRF4, which accelerates IRF4 protein degradation by proteasome. All these result in decrease of IRF4 level, leading to suppression of MM cell growth.

Our study uncovered a mechanism in which SUMOylation inhibition sensitizes Lenalidomide effects at decreasing IRF4 expression at transcriptional regulation of DOT1L and H3K79me2 and protein stability (Fig. 7). Unlike Len, which decreases IRF4 via CRBN-IKZF1/3 axis, TAK-981 decreases IRF4 transcriptional through epigenetic modulation at IRF4 promoter via downregulating DOT1L level. SUMOylation inhibition can decrease IRF4 level through enhancing protein degradation. Taken together, SUMOylation inhibition downregulated IRF4 at transcriptional and protein level, suppressed MM growth with overcoming Len resistance effect.

Epigenetic alterations including aberrant DNA methylation and histone modification play important roles in the pathogenesis of MM and are considered as potential therapeutic targets [36, 37]. Our previous work has reported SUMOylation regulates Enhancer of zeste homolog 2 (EZH2), the enzymatic component of polycomb repressive complex 2 (PRC2), which catalyzes trimethylation of histone H3 on lysine 27 (i.e., H3K27me3). SUMOylation inhibition caused decreased EZH2 and H3K27me3 level in colorectal cancer and breast cancer [27]. In this study, we demonstrated SUMOylation mediates another histone modification H3K79me2 via downregulating DOT1L. TAK-981 caused lower level of H3K79me2 on the promoter of IRF4 despite no change on H3K79me2 level in whole cell lysates, suggesting the regulation might be genome location specific. Interestingly, Len treatment also reduced DOT1L level but the effect was not abolished in Len-resistant cell line MMR10R, presenting a possibility that DOT1L might be involved in Len resistance mechanism.

We observed TAK-981 decreased EZH2 level in MM cells, consistent with our findings in colorectal cancer and breast cancer [27]. PRC2 activation and broad H3K27me3 formation was reported to promote MM tumorigenicity [38]. Taken together, the cytotoxicity effect of TAK-981 might be partially contributed by the downregulation on PRC2 and H3K27me3 level.

IRF4 activate c-Myc expression, and IRF4 was itself a direct target of MYC transactivation, generating an autoregulatory circuit in myeloma cells. In our previous work, we revealed SUMOylation regulates c-Myc mRNA level through regulation its targeting microRNA miR-34b/c [39]. We observed SUMOylation inhibition decreased c-Myc level, which might subsequently lower IRF4 level, leading to suppressed MM growth.

Together, our study revealed that SUMOylation inhibition enhances Len sensitivity in MM by decreasing IRF4 transcription level via downregulating DOT1L and IRF4 protein level via promoting degradation. Combination SUMO E1 inhibitor TAK-981 with Len showed potent synergistic anti-MM effects. We have also found SUMOylation inhibition enhanced MM sensitivity to dexamethasone, a synthetic glucocorticoid, which is most widely used in MM combination regimen. TAK-981 showed potent synergistic efficacy with dexamethasone against MM ex vivo and in vivo [34]. Since Lenalidomide plus dexamethasone is a standard of care for MM patients and resistance to the therapy remains a main challenge, our study revealed SUMOylation inhibition could be a novel strategy to address this need. Overall, our findings strongly support translation of TAK-981 into clinical trials for MM patients and possibly other hematologic malignancies with potential to improve outcome of the existing therapies.

## Supplementary information

supplementary-0114

## Data Availability

All data generated or analyzed during this study are included in this published article and its Supplementary Data files.
