# Supplementary material for: SUMOylation inhibition enhances multiple myeloma sensitivity to lenalidomide
Source: Cancer Gene Ther. 2022 Mar 25;30(4):567–74. doi: 10.1038/s41417-022-00450-9 (PMC10104776; doi:10.1038/s41417-022-00450-9)
Supplement: Supplementary file 1 — supplementary-0114 [file 41417_2022_450_MOESM1_ESM.pdf]

Title: SUMOylation inhibition enhances Multiple myeloma sensitivity to lenalidomide

Supplementary Material

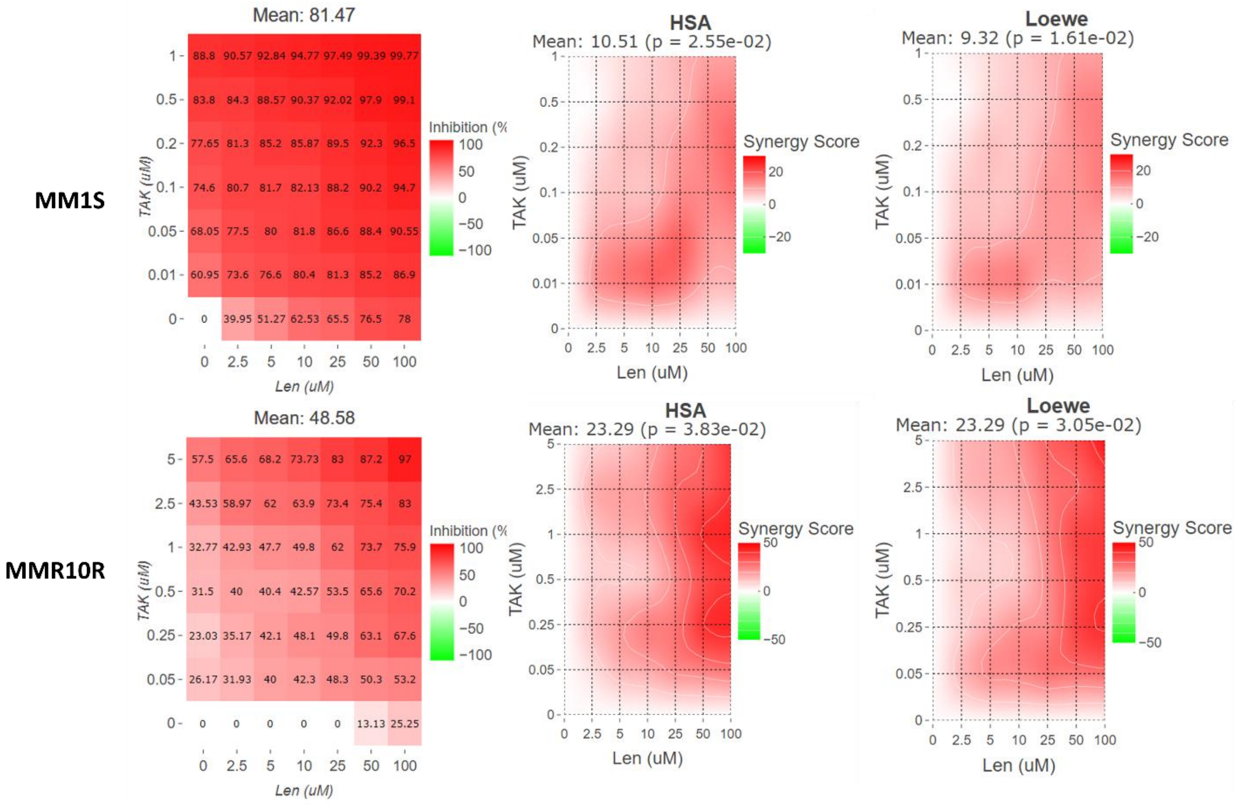

Supplementary Figure 1. Combinations of TAK-981 and Len show synergistic effects in drug-sensitive (MM1S) and –resistant (MMR10R) myeloma cells. MM1S (top) and MMR10R (bottom) cells were treated with indicated concentration of TAK-981 or Len or both (TAK+Len) with indicated concentration for 48 h and cell viability was determined by Cell-Titer-Glo. SynergyFinder software was used to calculate inhibition (left panel) and synergy scores using effect-based strategy, Highest Single Agent (HSA) model (middle panel) or dose-effect-based strategies, Loewe additivity model (right panel). Synergy scores > 0 indicate synergism (red regions) and scores < 0 indicate antagonism (green regions). One representative experiment of at least 3 is shown.

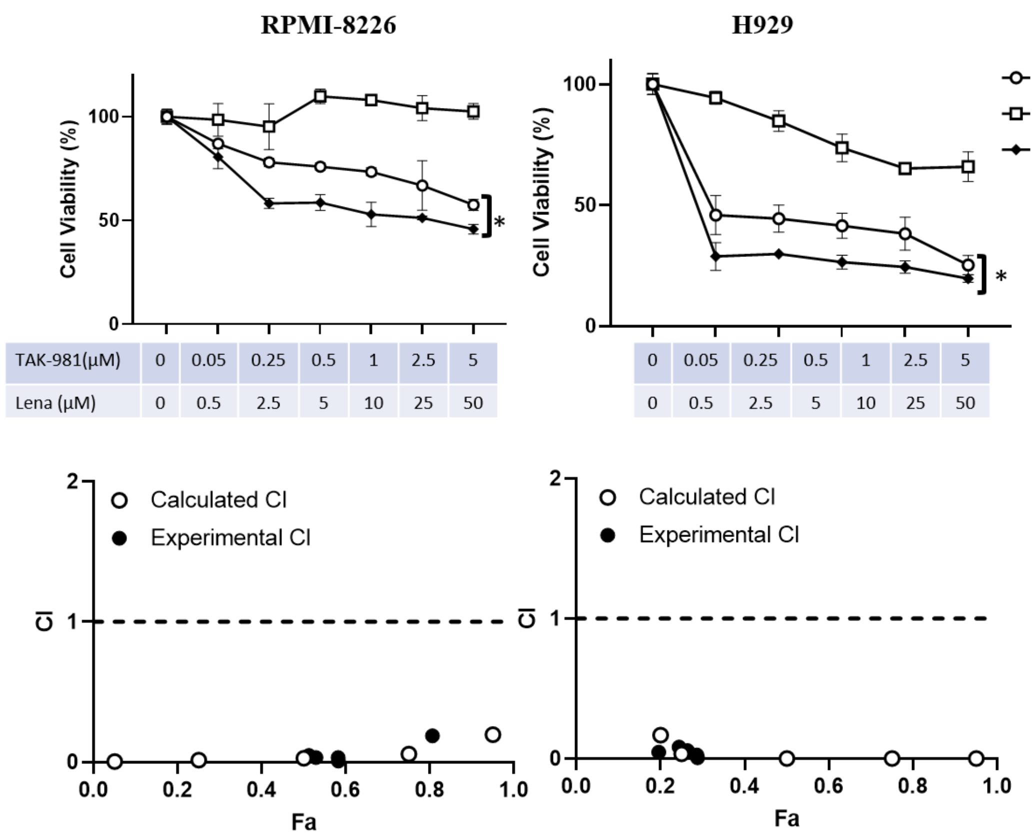

TAK-981(µM)	0	0.05	0.25	0.5	1	2.5	5
Lena (µM)	0	0.5	2.5	5	10	25	50

0	0.05	0.25	0.5	1	2.5	5
0	0.5	2.5	5	10	25	50

CI	ED50
MM1S	0.0311
MMR10R	0.1870
RPMI8226	0.0279
H929	2.64E-4

Supplementary Figure 2. Cell viability assay of RPMI-8226, H929 and KMS11 cell lines with TAK-981 and Len treatment. RPMI-8226, H929 and KMS11 cells were treated with indicated concentration of TAK-981 or Len or both (TAK+Len) with indicated concentration for 48 h and cell viability was determined by Cell-Titer-Glo post-48h treatment. Drug synergy was analyzed using CompuSym program. Simulating calculated CI values (open circle) and experimental combination indice (CI) values (solid circle) based on combination data points are plotted as a function of the fractional affected (Fa) derived from analysis report (bottom panel). Drug synergism is defined as CI < 1. CI values at effect doses (ED50) were listed.

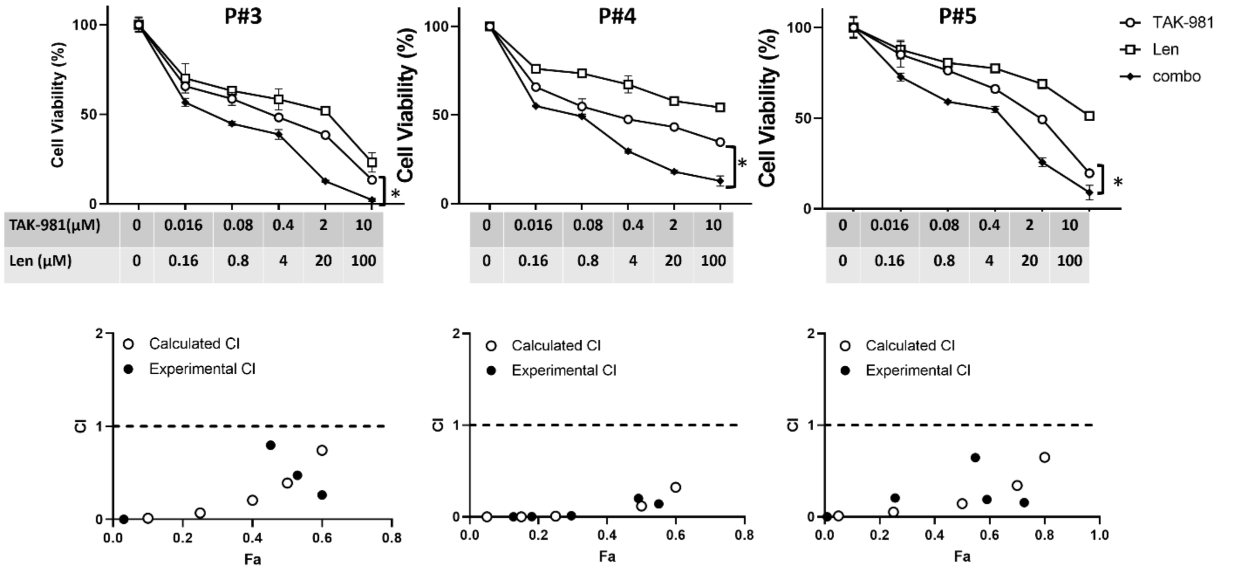

P#1		P#3		P#4		P#5			
**Fa**	**CI**	**Fa**	**CI**	**Fa**	**CI**	**Fa**	**CI**		**CI @ED50**
0.4765	0.060	0.6	0.263	0.5505	0.141	0.726	0.156	**P#1**	0.1562
0.4455	0.190	0.529	0.474	0.4915	0.201	0.59	0.190	**P#3**	0.3906
0.3455	0.214	0.452	0.798	0.296	0.012	0.548	0.646	**P#4**	0.1177
0.23	0.149	0.384	0.488	0.1805	0.002	0.2565	0.206	**P#5**	0.1447
0.0795	0.011	0.031	0.000	0.1275	0.001	0.0089	0.000		

Supplementary Figure 3. SUMOylation inhibition synergizes with Len in decreasing cell viability in primary multiple myeloma cells. Cell viability assay showing 3 primary CD138+ cells from bone marrow aspirates of relapsing MM patients treated with TAK-981 or Len or both (TAK+Len) with indicated concentration. Cell viability was assessed by Cell-Titer-Glo after 48 hours of treatment. Drug synergy was analyzed using CompuSym program. Simulating calculated CI values (open circle) and experimental combination indice (CI) values (solid circle) based on combination data points are plotted (middle panel) as a function of the fractional affected (Fa) derived from analysis report (bottom panel). Drug synergism is defined as CI < 1. CI values at effect doses (ED50) were listed.

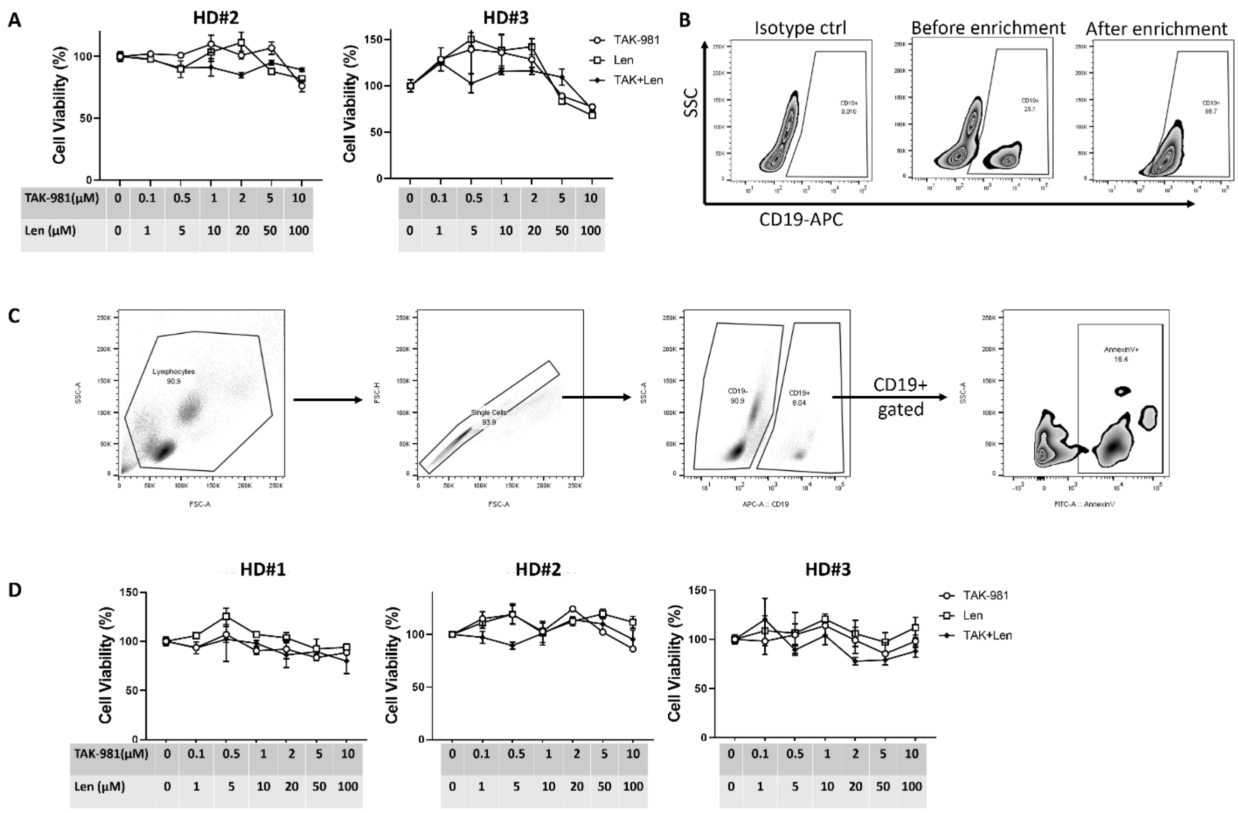

Supplementary Figure 4. TAK-981 or Len or both showed no effect on normal B lymphocytes cell viability from healthy donors. (A) Cell viability assay showing 2 primary CD19+ cells from PBMCs of healthy donors (HD). Normal B lymphocytes were purified by Mojosort human CD19+ cell selection kit. CD19+ cells were treated with TAK-981 or Len or both (TAK+Len) with indicated concentration. Cell viability was assessed by Cell-Titer-Glo after 48 hours of treatment. (B) Flow cytometry shows the purity of normal B lymphocytes enrichment. CD19 staining of PBMCs before and after enrichment by Mojosort selection kit. (C) Gating strategy for main Figure 3C. (D) TAK-981 or Len or both showed no effect on cell viability of health donor PBMCs. Cell viability assay showing PBMCs from 3 healthy donors treated with TAK-981 or Len or both (TAK+Len) with indicated concentration. Cell viability was assessed by Cell-Titer-Glo after 48 hours of treatment.

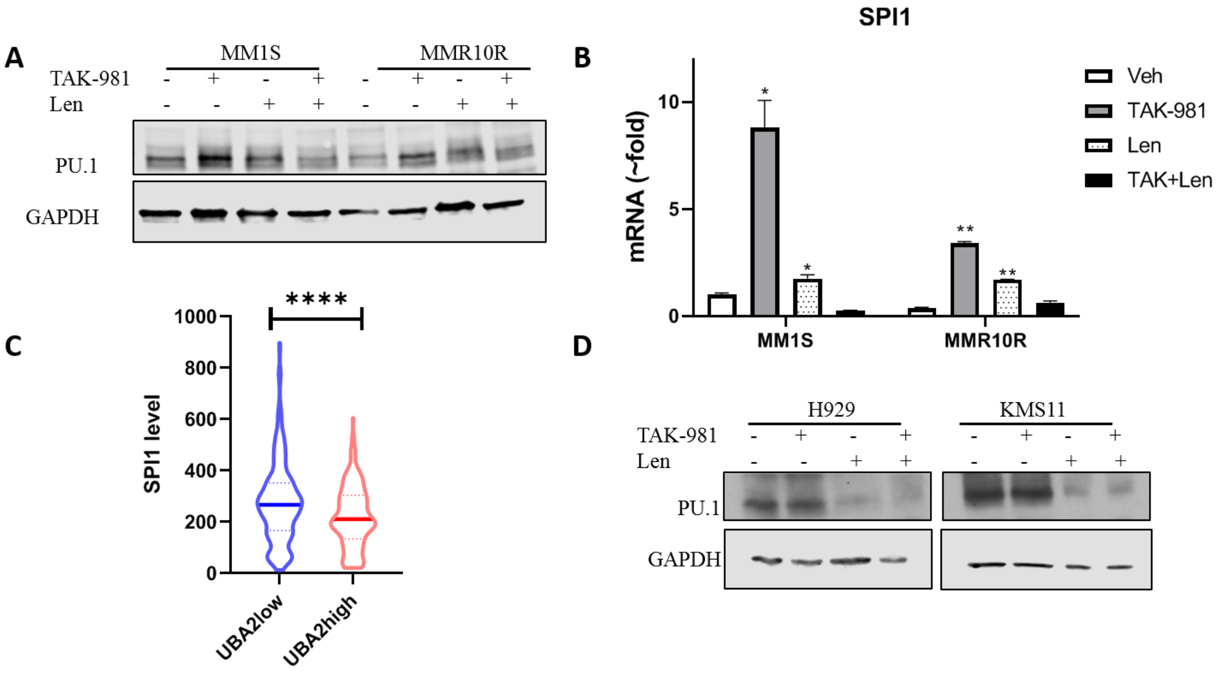

Supplementary Figure 5. The effects of SUMOylation inhibition on PU.1 expression in MM. (A) Western blotting of PU.1 protein level and (B) qPCR of PU.1 (SPI1) mRNA level upon TAK-981 or Len or both treatment in MM1S and MMR10R cell lines. (C) UBA2 level negatively correlates with SPI1 expression in patient specimens. Analysis of cohort (GSE2658) of 559 MM patients. Patients with high SAE2 (UBA2; UBA2high group) showed lower SPI1 level than patients with low SAE2 (UBA2; UBA2low group). Data were analyzed using unpaired Student t tests: Data presented as mean±SD. ****, $p<0.0001$. (D) Western blot showing PU.1 level upon TAK-981 or Len or both treatment in H929 and KMS11 cell lines. MM1S, MMR10R and H929 cells were treated with TAK-981 (0.1 µM) or Len 2.5 µM or both for 48 h; KMS11 cells were treated with TAK-981 (1 µM) or Len (25 µM) or both for 48 h. GAPDH, loading control.

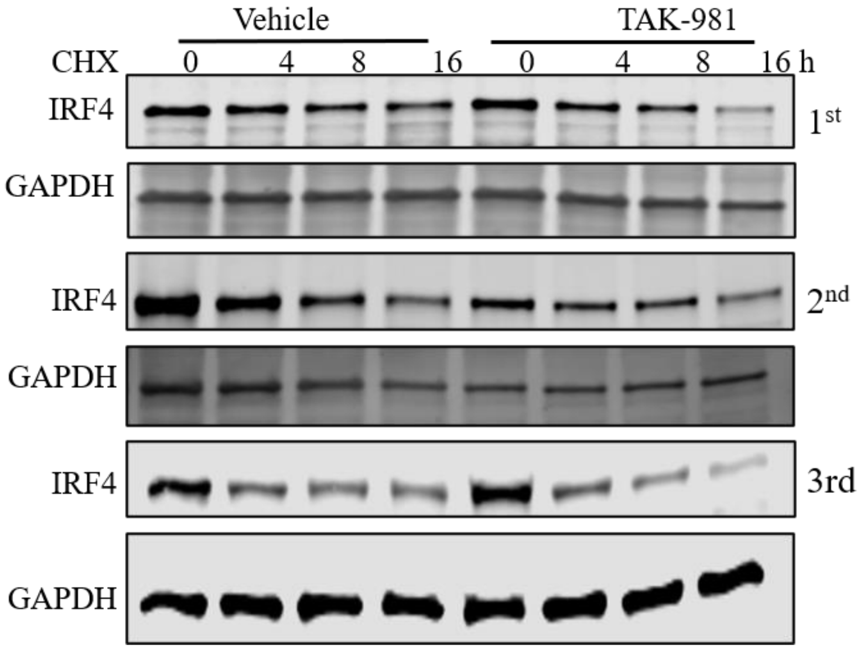

Supplementary Figure 6. SUMOylation inhibition accelerates IRF4 protein degradation. Representative western blot of IRF4 level over time in MMR10R cells treated with or without TAK-981(0.1 μM) for 4 h, followed by 100 μg ml^{-1} CHX treatment for indicated time; GAPDH, loading control. Results of three independent experiments were shown.
